# Organizational Culture Relation With Innovation: Comment on "Employee-Driven Innovation in Health Organizations: Insights From a Scoping Review"

**DOI:** 10.34172/ijhpm.8583

**Published:** 2024-09-07

**Authors:** Hatem H. Alsaqqa

**Affiliations:** ^1^Deanship of Research, Al-Quds University, Gaza, Palestine.; ^2^Ministry of Health, Gaza, Palestine.

**Keywords:** Behavior, Health, Innovation, Organizational Culture, Strategy

## Abstract

This commentary article is important and relevant, as organizational culture and innovation are essential to health organization’s performance and viability in a dynamic and competitive setting. Organizational culture is the unquenchable drive that propels an organization’s growth and serves as its soul. However, effective internal innovation management is one way that managers and organizations may foster innovation. Health organizations that prioritize service and technological innovation while simultaneously cultivating an appropriate innovation culture to establish a sustainable internal consensus that stimulates innovation are the most inventive. Individuals as well as groups have a deeply ingrained culture that influences how they think and behave, causing health organizations to operate in a way that is rational and consistent.

 Employee-driven innovation focuses on the influence that a group of healthcare providers, healthcare workers, and administrators of healthcare organizations have on the rise of health innovations.^1^

 The intricate network of shared beliefs, commitments, customs, and values that permeates an organization and shapes how it operates as a whole is known as its organizational culture. Then, this culture can be a source of innovation. Culture is defined as the common set of values, conventions, presumptions, and beliefs that influence how members of an organization behave and perform. Among many other elements, organizational culture is one of the most important features that affects an organization’s capacity for innovation. Therefore, one of the main reasons for the interest in organizational culture research is the claim or presumption that organizational cultures produce better organizational outcomes.

 Though innovation is vital for health organizations in attaining their long-term goals in light of the rapid shifts occurring in the modern world, a dynamic and innovative culture generates the ideal conditions for the blooming of creativity and teamwork. By fostering a collaborative, resource-rich, and sharing-knowledge setting, an organizational culture encourages staff members to fully utilize their strengths. This positively impacts staff’s creativity, self-efficacy, and intrinsic drives and encourages them to act in innovative ways.

 Kenny and Reedy^2^ argue the belief that “innovative organizational culture is one in which continuous improvement throughout the organization is the norm” (p. 119). Innovation is a mentality that permeates the entire organization and is shared by all personnel. The same holds true to organizations where personnel need to be creative thinkers in order to improve overall performance and deliver superior performance or services.

 The organizational culture and innovative behavior of employees in health organizations are closely related. The organizational culture establishes the values and norms of behavior within the health organization, facilitating employees’ better integration into the workforce and enhancing productivity. Additionally, employees’ behavioral traits can be controlled and modified in accordance with the health organizational culture’s activities, further fostering staffs’ better integration into the organization and fostering mutual understanding and growth between both staff and managers.^3^

 Therefore, an adaptive health organization cultivates an innovative culture and is always looking for behaviors to improve. It motivates staff members to question the status quo, investigate novel concepts, and try out creative approaches. An innovative culture facilitates staffs’ acquisition of new skills, knowledge, and competences to meet changing demands. However, staff mindsets are significantly impacted by new work organization strategies, particularly when it comes to managing creative behaviors. This includes a shift in how they view the meaning of innovations for the health organization and how they value teamwork.^4^

 When the organizational culture is more encouraging to innovation in health organizations, it is possible to increase employees’ intrinsic motivation, which will encourage them to act in more creative ways. This is due to the fact that an organization’s culture fosters their sense of autonomy, accomplishment, and inquisitiveness.^3^ This approach views organizational culture management as a means for more significant organizational change, or organizational innovation, and as such, it forms a significant part of the health organization’s inventive behavior growth.^5^

 Nevertheless, culture affects individual exertions, group actions, and internal health organization views and behaviors regarding opportunities and risks. These factors all have an effect on models of innovation and invention. The primary measure of innovation effectiveness is innovation quality, which takes into account the novelty and inventiveness of new concepts, goods, procedures, methods, and organizational management.^6^

 However, the health sector has a great deal of conceptual variance. Private, public, and other sectors may all have different viewpoints on this issue. These days, it is important to draw attention to a developing and significant idea called “responsible innovation.”^7^ The goal of the research on responsible innovation in the health sector is to emphasize how crucial it is to match the values of society with the processes and outcomes of innovation. But as organizations aim to employ strict standards in the recruitment, selection, and investment of persons, they also need to understand that organizational elements can either stimulate or impede the creative potential of both the individual and the group.

 Nonetheless, a number of researchers acknowledge a complex dynamic equilibrium necessitating that health organizations have a strategy tailored to every situation. This strategy should enable the formation of different initiatives (sometimes bottom-up, sometimes top-down), with management backing that provides time for introspection, experimentation, and quality evaluation standards associated with this inventive and creative ability. Furthermore, outside variables like government regulations, funding from the public and private sectors, and the pursuit of external legitimacy may foster and improve innovation in health organizations.^7^

 Furthermore, for health organizations’ innovation strategy to be implemented effectively and efficiently, organizational culture and innovation strategy have to simultaneously coexist.^6^ The determination to be innovative, the infrastructure to sustain innovation, the operational level behaviors required to influence the setting and value orientation, and the environment to implement innovation, are all factors that contribute to the multifaceted context of innovation in health organization’s culture. Positivity in the organization’s culture provides the necessary components for innovation and can be assimilated by dynamic organizations into their management procedures and organizational culture.^8^

 As Schein and Schein^5^ defined organizational culture; this is known as “Schein’s model of organizational culture,” and it highlights the layers or levels of culture, which comprise artifacts, behavioral patterns, norms, and values. The earlier research by Hogan and Coote^9^ tested the applicability of Schein’s multi-layered organizational culture model for considering procedures that promote innovation. Different types of organizational cultures also have a significant impact on the innovative behavior of staffs.

 The Competing Values Framework (CVF), which is a predictor of outcomes like quality improvement, and patient and professional satisfaction, has been frequently employed in studies and research to examine the culture of health organizations. One of the most well-known and heuristic conceptual frameworks for integrating the key components of organizational “effectiveness” is called the CVF.^10^ It is a composite of ideas about organizations that describe them in two ways: (1) flexibility – stability and control; (2) internal environment – external environment. These theories each offer a different way of looking at fundamental problems that organizations need to overcome in order to operate. The idea is that these four cultures are archetypes. Organizations are required to exhibit elements of all four cultures. There is a dominant culture, but all four organizational cultures can coexist in a particular organization and do so fairly steadily over time, according to CVF.

 Furthermore, high convergence cultures in health organizations encourage both internal integration and external adaptation, which in turn influences staff innovation behavior. Group cohesion and integration are greatly influenced and valued by organizational culture.^3^ According to many findings, organizational innovativeness and three different organizational culture types—adhocracy, market, and clan—have a strong positive correlation. Furthermore, the adhocracy culture has the largest contribution to the prediction of organizational innovativeness, despite the hierarchy culture showing a non-significant link with it.^8^

 Staffs in high convergence cultures—where the emphasis is on both outward adaptation and internal integration—are more driven to act creatively and are more confident in their abilities. This is due to the fact that this cultural pattern prioritizes stability and interpersonal harmony while attempting to balance the interests of all parties. Additionally, this culture places a strong emphasis on staff orientation on the inside, fostering democratic involvement and staff loyalty, and social responsibility and the bravery to innovate on the outside.^3^

 Adhocracy culture prioritizes adaptability and is outwardly focused, emphasizing risk-taking, inventiveness, and entrepreneurship. Clan culture is inwardly concentrated and flexible. Teamwork, employee engagement, and identity dedication to employees are traits of organizations with this culture. Market culture is outwardly focused and control-oriented. Although there is a low degree of trust, morale, and change resistance in this culture, productivity and competition are valued. Internal attention and control are hallmarks of hierarchical culture. This culture places a strong emphasis on efficiency, standardization, and strict conformity to laws and guidelines.^6^

 Contradictory, managers in high-power distance cultures could be less inclined to give up control and more likely to utilize their position of authority to influence subordinates. Managers can gain a deeper grasp of the relationships and tensions between various forms of innovation strategy and organizational culture by utilizing the fit perspective. This proposes that when managers successfully apply an exploitative innovation strategy, they should control clan culture and foster hierarchical culture. Meanwhile, we propose that nurturing an adhocracy culture will facilitate the execution of an exploratory innovation approach.^6^

 Twenty-one of the 44 papers that were assessed looked into the elements that influence innovation readiness during the innovation process’ implementation stage. The stages of concept generation, idea selection, solution development, implementation, scale-up, and diffusion are considered to be the essential components of a comprehensive innovation process.^11^ In the early phases, opportunities for innovation are sought, chosen, and developed. During the final phase, steps are implemented to facilitate the widespread adoption of innovation within the organization.

 However, the emphasis on an evidence-based approach in healthcare may have stimulated an impulse for innovation to be implemented. Ten sub-factors that were grouped into four primary factors—strategic course for innovation, climate for innovation, leadership for innovation, and commitment to innovation—that together accounted for the innovation readiness of healthcare organizations were identified by research.^11^ Feeling informed, change appropriateness, and change efficacy were found to have positive and significant connections with collaboration culture and change management factors.

 In order to positively impact organizational culture and increase the likelihood of a seamless change transfer, staff members must also believe that the change is appropriate and that they can handle it. The organizational change literature has given readiness for change a lot of attention because it plays a crucial role in the success of organizational transformation.^12^ Because of their advocacy and collaborative power (ie, bridging barriers and passing on knowledge), change agents are critical to the success of organizational change.

 The literature suggests that the primary element of innovation strategy is the proportionate focus on exploratory vs exploitative innovation.^13^ Yet, numerous research studies have demonstrated that it is hard to achieve high identity sustainability and robust innovation without modifying social dynamics, updating networking and communication tactics within organizations, and modifying value systems and knowledge.^14^ If organizations want to generate continuous and long-lasting value, they must develop and adopt an innovation culture that supports the development of the capabilities needed to succeed competitively in the present and the future.

 Furthermore, a sample of 192 hospital administrators was asked about the culture of their organizations, how well they could adopt new technology, and how successful they thought their most recent information technology implementation was after it had been in place for at least a year. The findings highlight the significance of organizational culture in both the development of absorptive ability and its impact on the adoption of new technology.^15^ A culture that emphasizes improvement and keeps an eye outwardly focused on the competitive landscape, would foster knowledge development and the use of communication channels to disseminate that knowledge.

 Nonetheless, researchers realize that health organizational innovation is complicated and influenced by a range of internal and external influences, but they also recognize that not all cultural values have the same effect on innovation. Innovative activities are supported by values that include taking risks, being creative, independence, autonomy, flexibility, adaptability, and acceptance of mistakes.

*In summary,* modern management theory ought to incorporate organizational culture as a theoretical idea and its practical implementation in the context of health organizations. In order to guarantee that the health organization’s efficiency is attained, organizational culture formation aids employees in becoming acquainted with the organization, controlling their behavior in compliance with the values of the organization, and responding promptly in accordance with the organization’s strategy.

 As innovative behavior improves performance, creates, encourages, and applies new thought within the organization, it allows staff members to promptly and accurately adapt, and using their innovative mentality to changes in health services needs.^3^ Health stakeholders may better comprehend the dynamics of innovation processes, pinpoint opportunities for development, and create strategies that effectively foster both innovation and organizational growth by having a thorough awareness of organizational culture.

## Ethical issues

 Not applicable.

## Conflict of interests

 Author declares that he has no conflict of interests.
